# Deep‐learning‐assisted algorithm for catheter reconstruction during MR‐only gynecological interstitial brachytherapy

**DOI:** 10.1002/acm2.13494

**Published:** 2021-12-10

**Authors:** Amani Shaaer, Moti Paudel, Mackenzie Smith, Frances Tonolete, Ananth Ravi

**Affiliations:** ^1^ Department of Physics Ryerson University Toronto Ontario Canada; ^2^ Department of Biomedical Physics King Faisal Specialist Hospital and Research Centre Riyadh Saudi Arabia; ^3^ Department of Medical Physics Sunnybrook Health Sciences Centre Toronto Ontario Canada; ^4^ Department of Medical Physics University of Toronto Toronto Ontario Canada; ^5^ Department of Radiation Therapy Sunnybrook Health Sciences Centre Toronto Ontario Canada

**Keywords:** catheter reconstruction, deep learning, gynecological brachytherapy, HDR, MR‐only brachytherapy

## Abstract

Magnetic resonance imaging (MRI) offers excellent soft‐tissue contrast enabling the contouring of targets and organs at risk during gynecological interstitial brachytherapy procedure. Despite its advantage, one of the main obstacles preventing a transition to an MRI‐only workflow is that implanted plastic catheters are not reliably visualized on MR images. This study aims to evaluate the feasibility of a deep‐learning‐based algorithm for semiautomatic reconstruction of interstitial catheters during an MR‐only workflow. MR images of 20 gynecological patients were used in this study. Note that 360 catheters were reconstructed using T1‐ and T2‐weighted images by five experienced brachytherapy planners. The mean of the five reconstructed paths were used for training (257 catheters), validation (15 catheters), and testing/evaluation (88 catheters). To automatically identify and localize the catheters, a two‐dimensional (2D) U‐net algorithm was used to find their approximate location in each image slice. Once localized, thresholding was applied to those regions to find the extrema, as catheters appear as bright and dark regions in T1‐ and T2‐weighted images, respectively. The localized dwell positions of the proposed algorithm were compared to the ground truth reconstruction. Reconstruction time was also evaluated. A total of 34 009 catheter dwell positions were evaluated between the algorithm and all planners to estimate the reconstruction variability. The average variation was 0.97 ± 0.66 mm. The average reconstruction time for this approach was 11 ± 1 min, compared with 46 ± 10 min for the expert planners. This study suggests that the proposed deep learning, MR‐based framework has potential to replace the conventional manual catheter reconstruction. The adoption of this approach in the brachytherapy workflow is expected to improve treatment efficiency while reducing planning time, resources, and human errors.

## 1 INTRODUCTION

The standard of care for locally advanced cervical cancer is to administer external beam radiation therapy with chemotherapy followed by brachytherapy.^1^, ^2^, ^3^ Interstitial high dose rate (HDR) brachytherapy is a crucial form of brachytherapy for improving the overall survival while limiting treatment toxicity in patients with larger tumors or asymmetric tumor morphology.^4^, ^5^, ^6^, ^7^, ^8^

The adoption of three‐dimensional (3D) imaging in the interstitial gynecological workflow using magnetic resonance imaging (MRI) offers unparalleled soft‐tissue contrast enabling the delineation of targets and organs at risk (OAR). MRI helps identify patients with unique morphological features that would benefit from an interstitial implant. Several catheters are implanted through a brachytherapy template during interstitial gynecological brachytherapy to enable optimal conformity to the cervical disease's unique morphology. MRI images are then acquired for treatment planning purposes.^9^, ^10^ While the target and OARs can be delineated in MRI, the dark void appearance of the catheters is a challenge for human planners to differentiate from air cavities and other anatomical regions similar in appearance. Computed tomography (CT) is currently used as an adjunct to MRI during interstitial gynecological brachytherapy to visualize the catheters. However, the CT/MRI workflow is prone to registration uncertainties, longer procedure times, and additional radiation exposure.^11^ The standard approach to planning begins with manually reconstructing the positions of the catheters on the images. This manual process is challenging, prone to human error, and time consuming.^11^, ^12^ A transition to MR‐only treatment planning is desirable and would reduce the risks associated with adjunctive CT imaging.

To date, there have been a number of studies aimed at automatically localizing and reconstructing plastic brachytherapy catheters. The use of active MR‐tracked stylets for catheter localization has been reported.^13^ Hrinivich et al. reconstructed ring‐shaped and oval applicators in MRI images through a model‐to‐image registration algorithm.^14^ Recently, the use of deep learning for catheter segmentation has been reported during brachytherapy procedures in CT,^15^ MRI,^16^ and US.^14^ Jung et al. proposed a deep‐learning assisted approach to reconstruct ring and tandem applicators in gynecological HDR brachytherapy.^17^ The proposed method was able to automatically reconstruct the applicator in approximately 15 s per case. Dai et al. investigated a deep learning‐based approach to automatically reconstruct multiple catheters in MRI images for prostate HDR brachytherapy treatment planning.^18^ Their model was trained using the manual catheter reconstruction offered by experienced physicists as a ground truth image along with the original MRI images. After the network was trained, MRI images of a new prostate cancer patient were fed into the model to predict the locations and shapes of all the catheters. They were able to detect all catheters from 20 patients receiving HDR brachytherapy with a catheter tip error of 0.37 ± 1.68 mm. These findings confirmed that deep learning can successfully help with the development of catheter reconstruction during HDR brachytherapy.

The primary aim of this study was to evaluate the accuracy of a novel deep learning‐assisted semiautomatic algorithm to reconstruct interstitial catheters during MR‐only interstitial gynecological brachytherapy. We report the differences in catheter reconstruction between the algorithm and manual ground truth and the required time for catheter reconstruction.

## 2 MATERIALS AND METHODS

### 2.1 Data collection

The development of the reconstruction algorithm was based on MRI scans of twenty gynecological cancer patients treated with interstitial brachytherapy between 2018 and 2020 at the Odette Cancer Centre (ON, Canada). The local research ethics board approved the study. Patient characteristics are summarized in Table 1. Local standard of care MRI sequences were used for all the images on the same scanner; these included 3D T1 weighted (3D T1W) and 3D T2 weighted (3D T2W) images obtained using a standardized exam sequence on a 1.5 T (T) Ingenia MRI scanner (Philips Medical System, AMS). MRI scans were acquired with an in‐plane pixel size of 0.5327 × 0.5327 mm^2^ and a slice thickness of 1 mm. Three brachytherapy radiation therapists and two medical physicists with more than 100 patient cases experience manually reconstructed 360 catheters using the Oncentra Brachytherapy treatment planning system v.4.5.2 (Nucletron, Elekta AB, Stockholm, Sweden). Interobserver variability in catheter positions between the planners was evaluated in our previous study and found to be 0.68 ± 0.60 mm.^19^ Manual reconstruction was performed using both the 3D T1W and the 3D T2W MRI images, which provided complementary information to the planner during reconstruction. Markers were used to aid in the visualization of the catheters and have been previously described.^19^ Catheters containing these markers appear as signal voids (dark) on the 3D T2W images and as a positive signal (bright) on the 3D T1W images. The manual labels (*x* and *y* coordinates for each catheter) for each observer were averaged and used for training and testing the model. For training, the manual labels were used to identify the centroid of the catheter region within each of the raw image patches.

**TABLE 1:** Patient and tumor characteristics (*n* = 20)

Item	Number of patients (*n*)
Number of patients (*n*)	20
Median age (range) in years	62 (32–78)
Diagnosis	
malignant neoplasm cervix uteri	10
malignant neoplasm of endometrium	5
malignant neoplasm of vagina	5
FIGO stage	
IA‐IVA	5
IIB‐IIIB	7
Local recurrence	8
Intracavitary cases	4
Interstitial cases	16
Template type	Syed‐Neblett (*n* = 20)
Total number of catheters (mean ± SD)	360 (19 ± 4)
Number of fraction (median)	2

Abbreviation: FIGO, International Federation of Obstetrics and Gynecology.

### 2.2 System specifications

The proposed algorithm was implemented on a research computer running the 64‐bit Windows 7 Professional operating system (Microsoft, Redmond, Washington) with 16 CPUs processor (Intel, Santa Clara, California) and 16 GB of RAM. A GeForce GTX980 v.436.48 graphics card with 4 GB of memory was installed (NVIDIA, Santa Clara, CA). The proposed algorithm was implemented in Python (PyCharm v.2019.1.2, Python 3.5) using Keras with TensorFlow backend.^20^

### 2.3 The proposed semiautomatic catheters reconstruction algorithm

There are two steps in the proposed reconstruction algorithm. The first step was to segment the images to identify the suspected positions of catheters using the U‐net model.^21^ Two U‐net models were trained independently on T1W and T2W images, and probability masks of catheters were generated. In the second step, the generated probability masks were used to identify catheter positions on each slice through postprocessing steps. The following two sections will detail these two steps.

#### 2.3.1 U‐net network architecture

**FIGURE 1:**
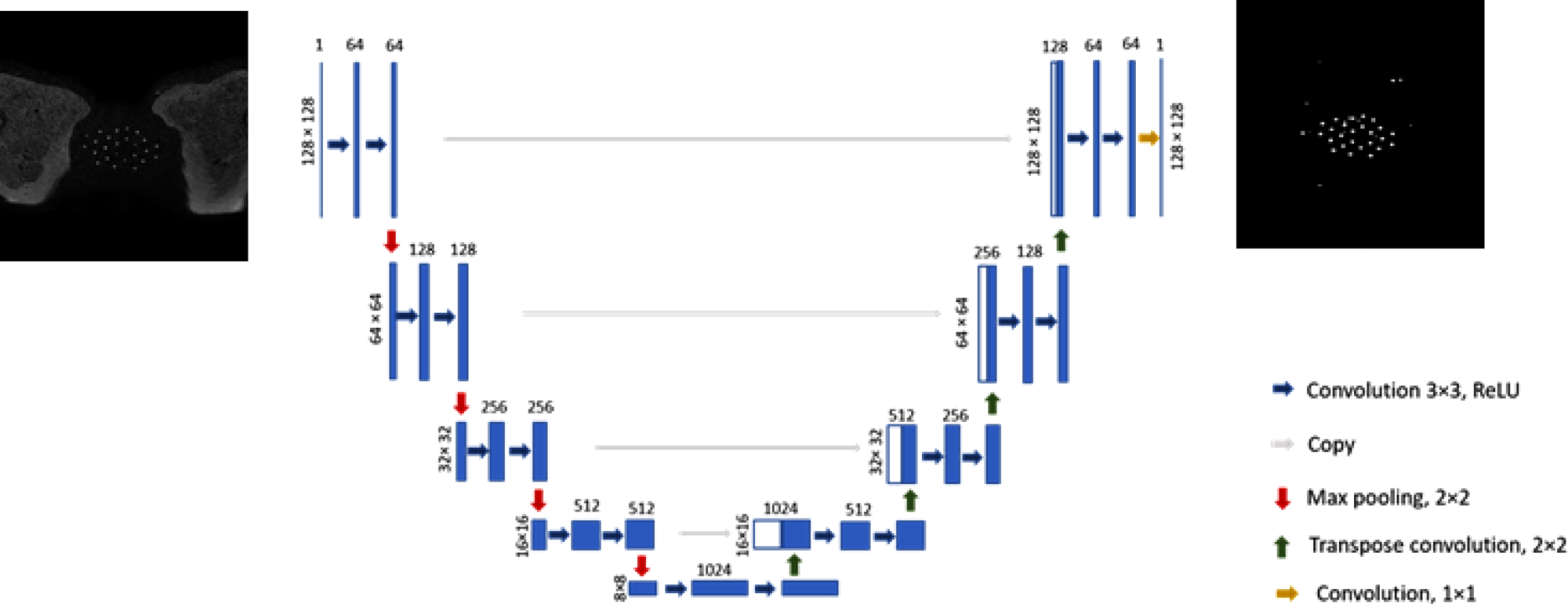
U‐net model architecture. The blue boxes show features maps. The number on top of each box represents the number of channels in each layer. The size of the input and output images were 128 × 128

Initial catheter segmentation was performed using a deep, convolutional neural network. Two 2D U‐net models were trained separately for 3D T1W and 3D T2W image sets. As shown in Figure 1, the U‐net model has symmetrical encoding and decoding parts. The encoding part involved five levels; each level was composed of two 3 × 3 convolution layers followed by a rectified linear unit activation function (ReLU)^22^ and a 2 × 2 max pooling, respectively. For this down‐sampling step, a stride size of 2 × 2 was applied to decrease the size of the feature maps from 128 × 128 to 8 × 8. The decoding part (up‐sampling) included four levels, each starting with a deconvolutional layer with a filter size of 2 × 2 followed by the ReLU function. Finally, a 1 × 1 convolution and sigmoid activation function were applied to generate probability masks.

The U‐net model was trained using the set of patients split into 70% (*n* = 14, 257 catheters) for training, 5% (*n* = 1, 15 catheters) for validation, and 25% (*n* = 5, 88 catheters) for testing.^23^ The model performance was improved using both T1W and T2W images perturbed by translation, scaling, and rotation in the axial plane. Perturbation is crucial to avoid training overfitting when a small training dataset is used. The training of the model was evaluated by quantifying the number of needles correctly identified after the model was trained using 1, 3, 5, 10, and 14 patients. This step was performed to illustrate how performance improved as a result of increased training data. A total of 41 547 64 × 64 patches were extracted from T1W and T2W images separately, which were then used to train the model. Both U‐net models were trained on the extracted 64 × 64 patches for 100 epochs with a batch size of eight. The number of epochs represents the number of times that the model will process the entire training set. The network was optimized by maximizing the Dice similarity coefficient between the predicted catheter locations and the ground truth provided by the planners (average of five planners). A small value ε was added to the numerator of the Dice equation (i.e., smooth) to avoid division by zero when both volumes, predicted and true, do not contain any foreground pixels. The learning rate was set to 1 × 10^–5^ with a He normal initializer. The total number of learning parameters was 31 031 685.

Training time was approximately one day for each of the T1W‐ and T2W‐based models, and the testing time was 1.5 s/slice (≈5 min per image volume). The network's output was a probability map, which quantifies the probability of each pixel in the patch as being a catheter or non‐catheter. Pixels with probability of more than 0.5 in the mask were considered as catheter regions, while pixels with probability of less than 0.5 were considered as non‐catheters regions.

#### 2.3.2 Post‐processing

**FIGURE 2:**
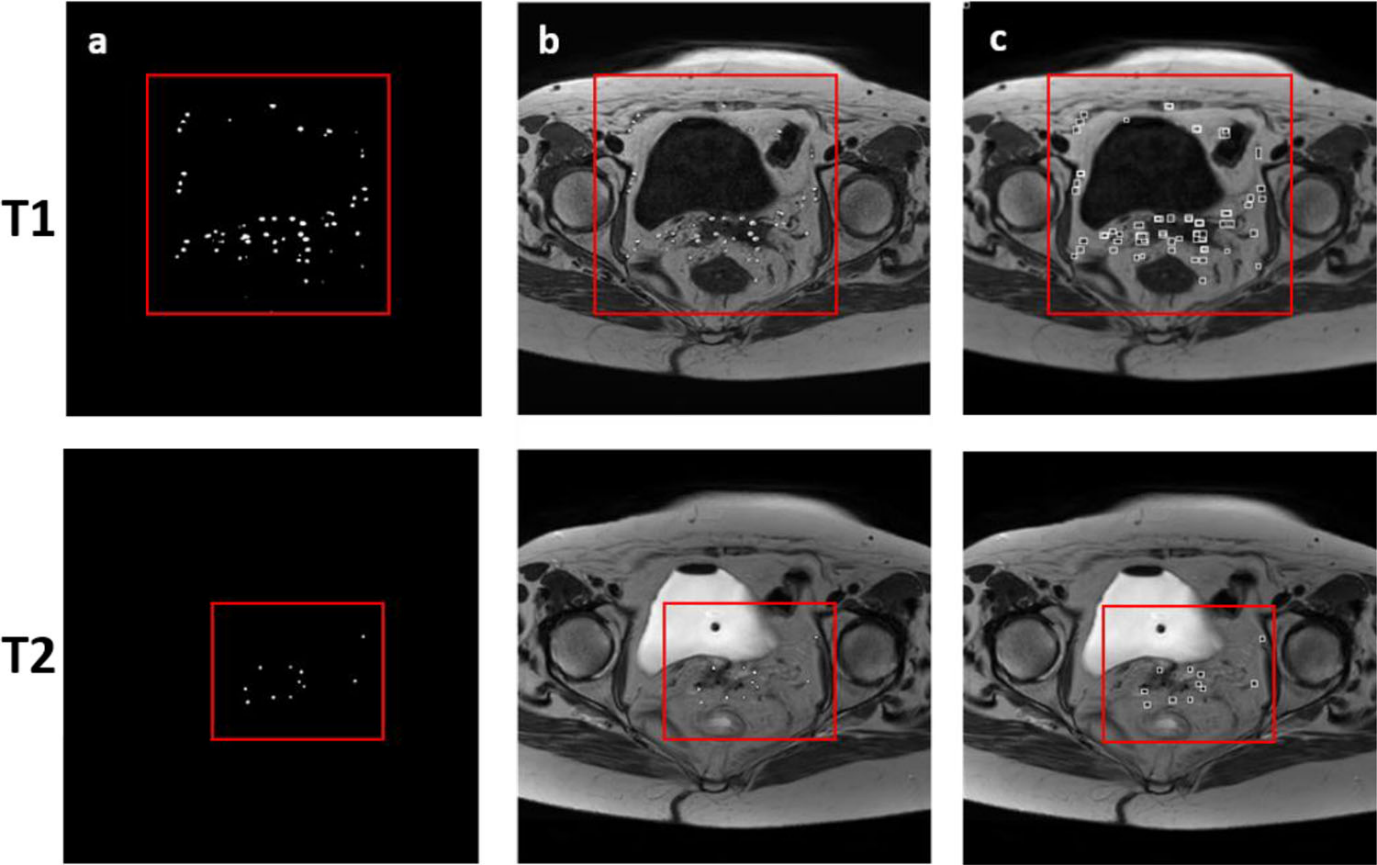
Algorithm postprocessing step using magnetic resonance imaging (MRI) images of one gynecological patient (testing set). (a) The red box shows the U‐net output (true and false positive catheter positions); (b) using the U‐net output approximate positions, catheters were located in the MRI images, T1w(top) and T2w (bottom) images, respectively; (c) rectangles were then calculated and used to find the exact positions of catheters in both image sets. As a final step, hyperintense (T1W) and hypointense (T2W) regions within those rectangular are localized as catheters

The second step of the proposed algorithm was to link segmented regions to reconstruct the catheter(s) within the images using the U‐net outputs (Figure 2). Using the approximate positions of the catheters, rectangles around those approximate positions were calculated (Figure 2c). The hyperintense (bright) and hypointense (dark) regions within those rectangles were labeled as catheters in the T1W and T2W images, respectively. The catheter position coordinates from the previous slice were used to eliminate the false positives and select the best catheters out of candidate labeled regions.

The ground truth slice (*m*) was selected, in which all the catheters were reconstructed from a starting plane (the inner plane of the physical trans‐perineal template that was in contact with the patient). In slice *m* + 1, the nearest candidate catheters to the ground truth (slice *m*) were selected as the true positives and the rest were eliminated. A physical free length measurement was used to stop the reconstruction of all catheters automatically. The free length (i.e., catheter length outside the patient) of the catheter along with the known thickness of the template were used to calculate the physical length of the catheter inside the patient (the total length of the catheter ‐ [free length + template thickness]). This step was necessary to ensure that all catheters were correctly detected and identified.

If segmented regions were not present within the 8‐pixel neighborhood centered on the location from the previous slice, the catheter was labeled a ``jumping catheter.” In these cases, the change in the positions (∆*x* and ∆*y*) of all correctly detected catheters was used to fix the position of the jumping catheter. We assumed that the positions of all predicted catheters in slice *n* were $\left\{\left(x_{1n},y_{1n}\right),\left(x_{2n},y_{2n}\right)\cdots \left(x_{in},y_{in}\right)\right\}$. In addition, the position of the same catheters in the following slice was $\{\left(x_{1(n+1)},y_{1(n+1)}\right)$, $(x_{2(n+1)}$, $y_{2(n+1)})\cdots (x_{i(n+1)}$, $y_{i(n+1)})\}$, where *i* represents the total number of catheters. Using this information, we can define the changes in the catheters’ positions as

$$\begin{array}{c} \Delta x=\frac{\left(|x_{1(n+1)}-x_{1n}|+|x_{2(n+1)}-x_{2n}|+\cdots +|x_{i(n+1)}-x_{\mathrm{in}}|\right)}{\text{No.}\;\text{of}\;\text{predicted}\;\text{catheters}} \end{array}$$

and

$$\begin{array}{c} \Delta y=\frac{\left(|y_{1\left(n+1\right)}-y_{1n}|+|y_{2(n+1)}-y_{2n}|+\cdots +|y_{i(n+1)}-y_{\mathrm{in}})\right|}{\text{No.}\;\text{of}\;\text{predicted}\;\text{catheters}} \end{array}$$

**FIGURE 3:**
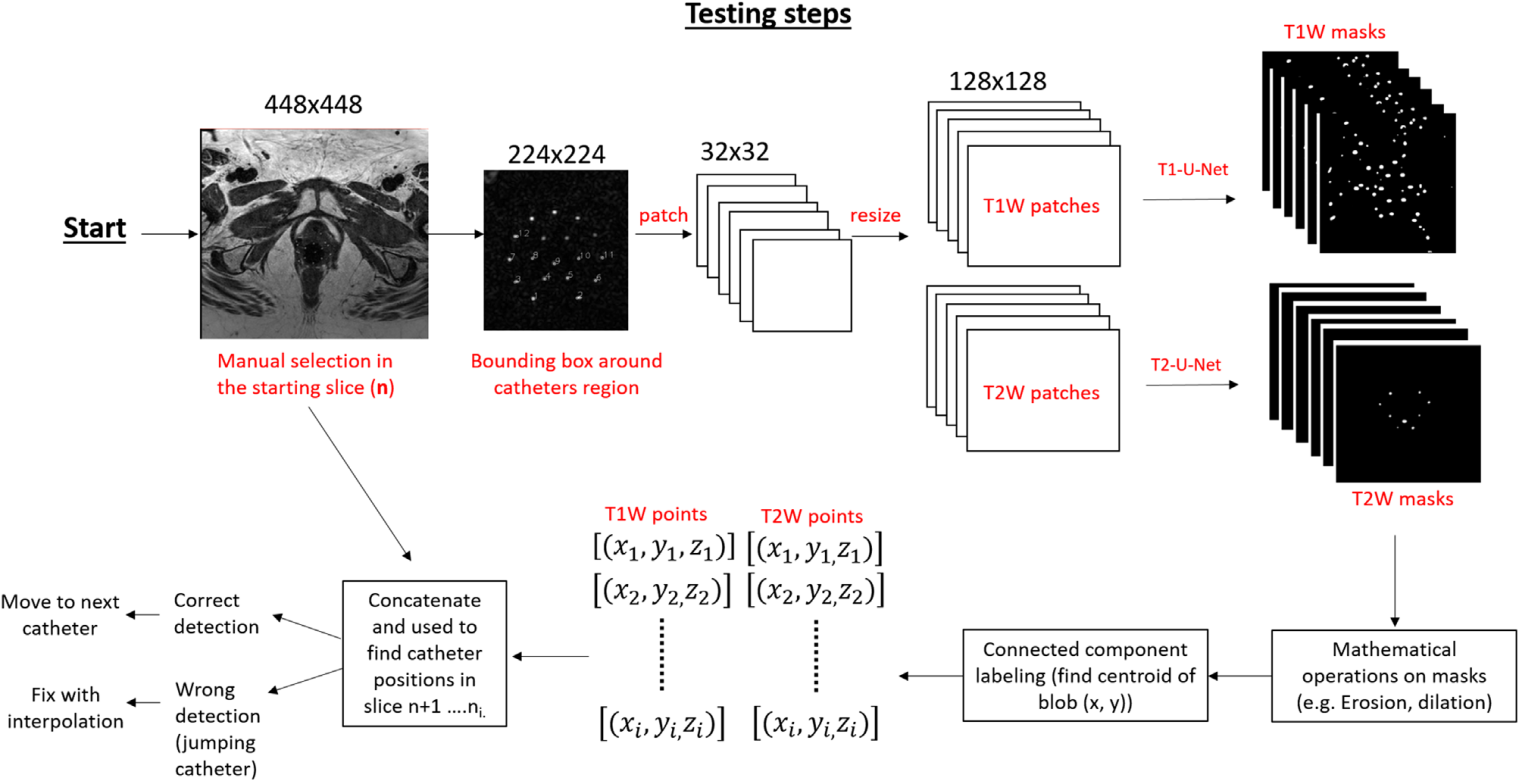
Workflow of the deep learning‐assisted algorithm for brachytherapy catheter reconstruction. The workflow starts with the manual selection of all catheters in the starting slice ***n*** (template plane) in T1W or T2W image. The images (both T1W and T2W) are then cropped and patched (size 128 × 128). These patches are then fed separately to two U‐Net models. Following, mathematical operations and connected component labeling are performed to accurately identify the centroid of the catheter region. The resulting catheters coordinates from T1W and T2W are concatenated and used to find the corrected location of the catheters in slices *n* + 1, *n* + 2….*n* + *i*, where *i* is the number of slices

The estimated Δ*x* and Δ*y* values were then added to the jumping catheter coordinates (*x* and *y*) to fix its position. By using this technique, the correct location of jumping catheters was calculated. Figure 3 illustrates the workflow of the semiautomatic algorithm.

## 3 RESULTS

### 3.1 Patient demographics

20 patients who underwent CT/MR‐based interstitial brachytherapy of either three or four fractions were enrolled in this retrospective study. The median age was 63 years (range, 32–78 years). The International Federation of Gynecology and Obstetrics (FIGO) stage distribution was IA‐IIIA (n = 5), IIB‐IIIB (*n* = 4) and IVA (*n* = 2). The remaining patients (*n* = 9) were treated for recurrent endometrial or vaginal carcinoma. All patients had template‐based interstitial brachytherapy using Syed‐Neblett template (Best Medical International Inc., Springfield, VA, USA) along with plastic catheters (ProGuide Sharp Catheter Set, 6F × 294 mm, Elekta, AB, Stockholm, Sweden). The results in this section are based on the testing dataset and averaged over five patients.

### 3.2 Training performance

**FIGURE 4:**
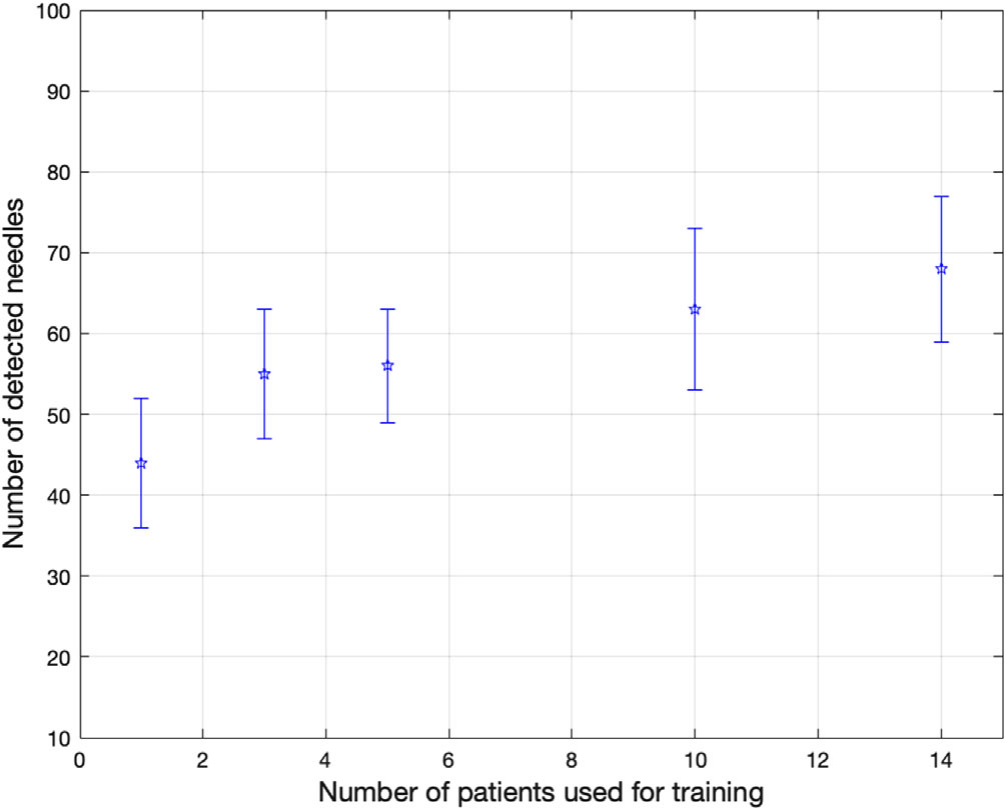
The impact of the number of patients used for training the UNet model on the number of needles correctly detected. Results shown were averaged over five patients (testing set). The number of patients used for training was 1, 3, 5, 10, and 14

The average Dice similarity coefficient (DSC) score was equal to 0.59 ± 0.10. Figure 4 illustrates the improvement in the performance of the algorithms as a result of training with datasets with incrementally larger sizes. Horizontal and vertical axes represent the number of patients in the training set and the number of needles correctly identified, respectively. Detection performance increases by adding more patients. Using 14 patients for training, the number of correct needles increases from 44 to approximately 66 needles (out of 88 needles in total).

### 3.3 Catheter reconstruction evaluation

**FIGURE 5:**
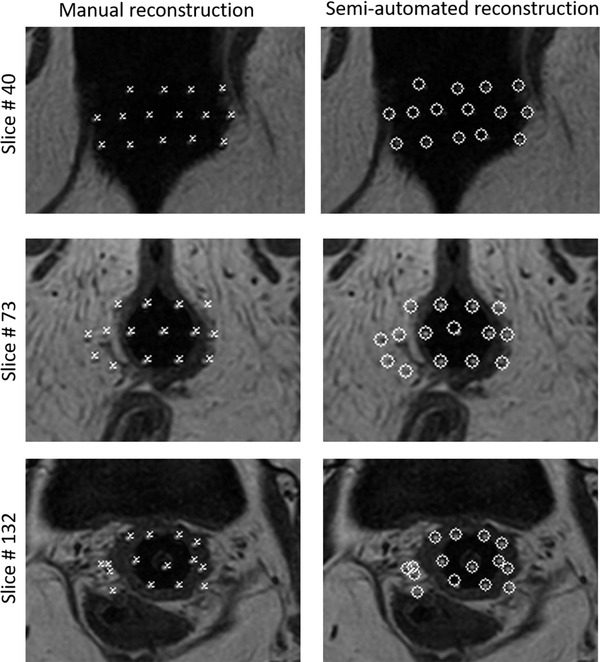
Axial T1‐Weighted MR image of a gynecological patient treated with 15 interstitial high dose rate (HDR) catheters. Left: manual reconstruction; (right) semiautomated reconstruction using the proposed algorithm

**FIGURE 6:**
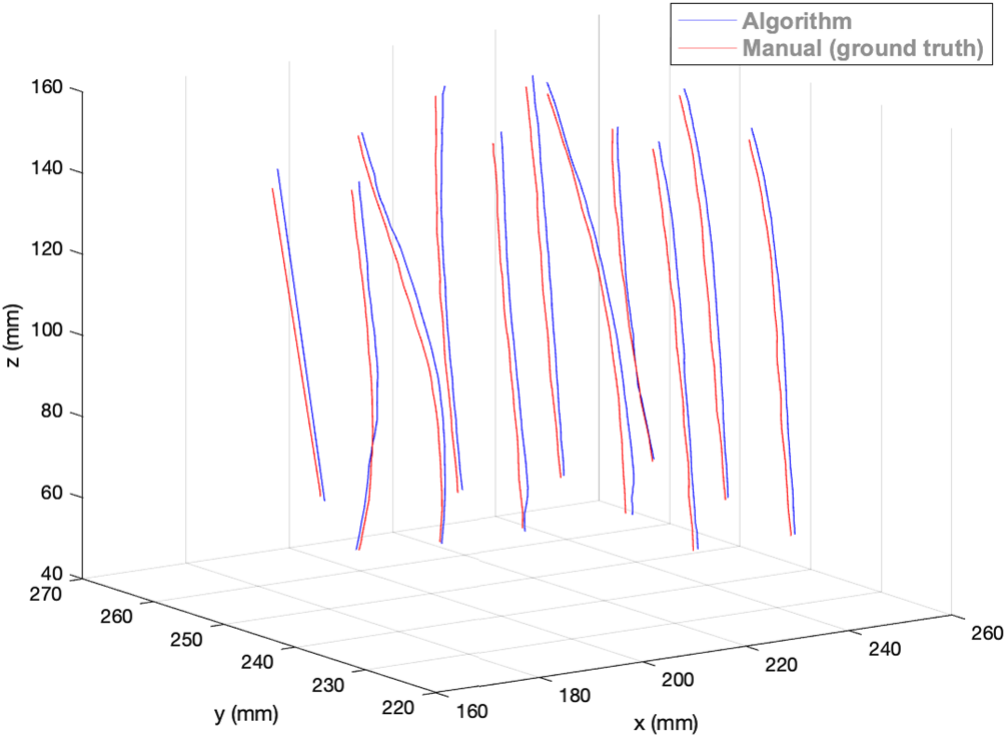
Reconstructed catheter paths obtained manually (red) and semiautomatically using the proposed algorithm (blue)

**FIGURE 7:**
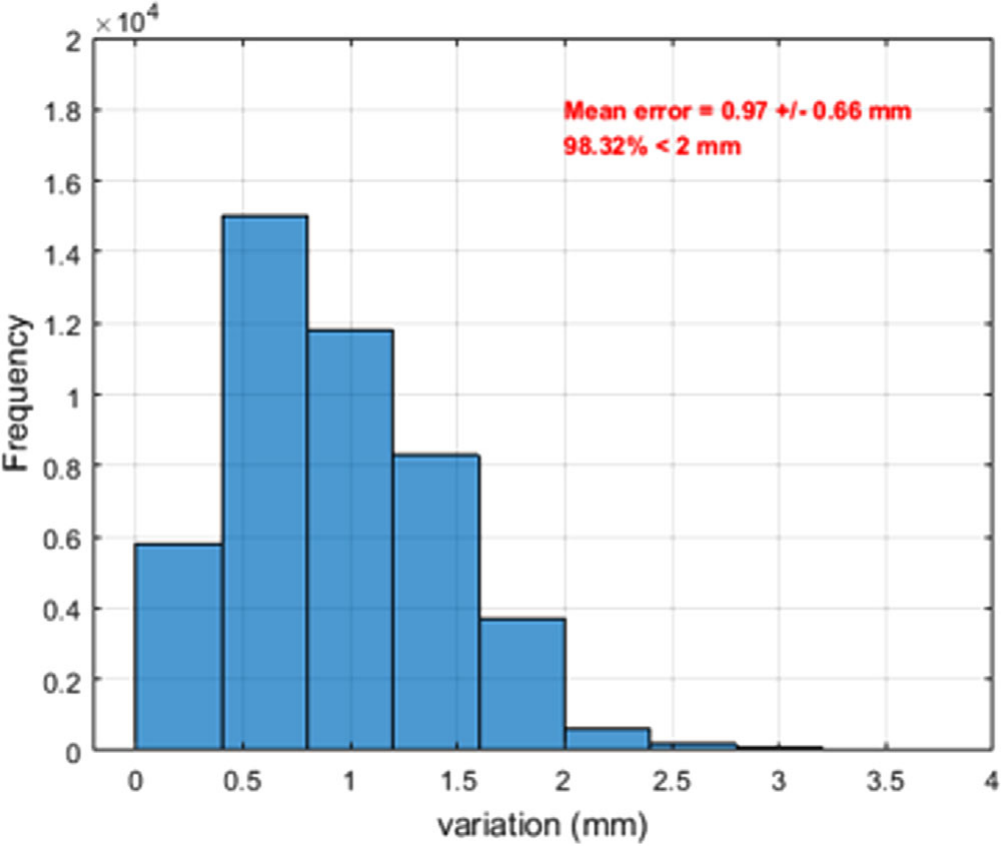
Line histogram shows the three‐dimension (3D) reconstruction variation between a manual and semiautomated algorithm in mm

Figure 5 shows axial T1W MR Images of a representative test case. No catheter was missed during the reconstruction process across all patients. Figure 6 illustrates semiautomated reconstruction (blue) and manual (red) catheter tracks for one gynecological patient. Figure 7 shows the variability in dwell positions between manual and semiautomated reconstruction presented as a linear histogram. A total of 34 009 catheter dwell positions, positioned at 1 mm intervals along the reconstructed catheter paths, were assessed over five patients to estimate reconstruction variability. The average variation was 0.97 ± 0.66 mm. More than 98.32% of dwell positions variations were < 2 mm; though, a few (1.68%) were more than 3 mm. Each catheter was visually tracked and reconstructed. A visual illustration in the axial view of one case is shown in the Supporting Information.

### 3.4 Catheter reconstruction time

The average reconstruction time across all five planners and patients was 46 ± 10 min. In contrast, the average time for the semiautomatic reconstruction was 11 ± 1 min, significantly lower (*p* < 0.001) than manual reconstruction.

## 4 DISCUSSION

There is a need to facilitate MR‐only gynecological brachytherapy workflows that eliminate CT imaging and may potentially enable a transformation into an outpatient procedure. MR‐based catheter reconstruction is, however, considered one of the main challenges in MR‐only interstitial brachytherapy. Implanted catheters are not easily visualized, and reconstruction is challenging. In this work, we proposed a novel deep learning‐assisted semiautomatic algorithm for catheter reconstruction during MR‐only interstitial gynecological brachytherapy treatment planning.

Accurate catheter reconstruction is necessary for MR‐only‐based gynecological interstitial brachytherapy. Systematic manual errors in reconstruction may result in significant uncertainties in dosimetric parameters for target and OARs.^11^ In this work, a semiautomatic algorithm was developed to replace manual reconstruction. A dataset of 360 catheters from 20 gynecological cancer patients was used to develop and test the proposed algorithm. The algorithm achieved a human planner level performance for the MRI‐based catheter reconstruction process. The results of the algorithm on an unseen test dataset showed an average variation in dwell positions of 0.97 ± 0.66 mm, which is clinically acceptable. According to GYN GEC‐ESTRO guideline, catheter reconstruction variation of more than 2 mm may lead to an undesirable impact on DVH parameters either for target or organs at risk.^2^

The algorithm proposed in this study was built to detect and reconstruct all catheters continuously in all slices using a two‐dimensional (2D) version of the U‐net model combined with post‐processing steps. In our approach, segmentation regions were predicted for the entire image volume by making predictions from previous slices. This work is partially dependent on the original form of the UNet deep learning model developed by Ronneberger et al.^21^ This model was chosen because (i) this model is based on the convolution neural network (CNN), which has been extensively used to develop automated accurate and stable detection and segmentation methods for the clinical target volume and brachytherapy catheters on US, MR and CT images during prostate and gynecological brachytherapy,^18^, ^24^, ^25^ and (ii) learned features from UNet CNN layers can be recognized regardless of their position in the image. This makes it useful for processing images with similar features (e.g., catheter positions), and is robust against variations in feature position or imaging conditions.^26^

Few studies have investigated the use of fully automatic or semiautomatic reconstruction of interstitial catheters during gynecological HDR brachytherapy.^27^, ^28^, ^29^ Most of these have developed catheter reconstruction methods for titanium applicators or are primarily based on CT images.^17^ The development of MRI‐based catheter reconstruction methods has been limited.^30^, ^31^ Of note is work by Zaffino et al., who conducted a study of 50 gynecological patients treated with MRI‐guided HDR brachytherapy with a total of 826 catheters.^30^ Using a deep 3D U‐net model, they achieved an average DSC of 0.60 ± 0.17, which is comparable to the value reported in this work (0.59 ± 0.10). They achieved an average Hausdorff distance of 2.0 ± 3.4 mm between manual and automatic reconstructions, which is less than the value reported in this study (4.20 ± 2.40 mm). 124 out of the 826 catheters (15%) were missed or incorrectly identified. The time needed to label a test MRI was 9 ± 2.5 min. A similar approach was implemented for catheter reconstruction during MR‐guided prostate brachytherapy.^18^ Other studies have explored the use of electromagnetic (EM) tracking based catheter reconstruction.^32^, ^33^, ^34^, ^35^ Poulin et al. have developed an EM tracking system for automated and real‐time catheter reconstruction in CT images.^32^ They reported a total reconstruction time of 3 min for a 17 catheter implant with a mean 3D distance error of 0.66 ± 0.33 mm.

The average reconstruction time of our semiautomated algorithm was <12 min per patient which makes a possible reduction in a clinical workflow time. Measured times include the manual selection of all the catheters at a reference slice. The elimination of the need for manual catheter reconstruction has the potential to improve reproducibility, safety and adoption of MR‐only for interstitial gynecological HDR brachytherapy.

There are some limitations of this study. First, the training step of the proposed algorithm was based on image patches. This might affect the efficiency of the algorithm since patch‐based training and testing are computationally slow compared to whole‐image‐based approaches which predict all pixel labels in one computation. Second, the number of test cases was limited; additional test cases are needed to further evaluate the algorithm's clinical utility. Third, the algorithm was developed and evaluated based on images using a standardized exam sequence of a 1.5 T MRI scanner to ensure that contrast in T1W and T2W images are maintained across the cohort of patients. The performance of the algorithm was not evaluated for other MRI scanners or image sequences. For sequences on different machines or even varying imaging settings, retraining of the U‐net model is recommended to ensure similar performance. Finally, the algorithm developed in this study is only valid for plastic interstitial catheters with or without MR line markers. This work was developed to ease the challenge associated with plastic catheter reconstruction during MR‐only gynecological workflow. It was not built to track or reconstruct tandem or metallic catheters. Note that retraining of the U‐net model would be required to reconstruct metal catheters.

## 5 CONCLUSION

A novel deep learning‐assisted semiautomatic algorithm for catheter reconstruction using MRI images was developed and evaluated in this study. The proposed algorithm was shown to be clinically feasible and accurate. This semiautomatic algorithm offers a unique opportunity to explore replacing the manual reconstruction of catheters during MR‐only interstitial gynecological HDR brachytherapy.

## CONFLICT OF INTEREST

The authors declare no conflict of interest.
